# The complete chloroplast genome sequence of a new variety of *Dendrobium officinale* ‘zhong ke IV hao’

**DOI:** 10.1080/23802359.2016.1219632

**Published:** 2016-09-02

**Authors:** Zhimin Zhong, Guifang Zhang, Xiaoping Lai, Song Huang

**Affiliations:** a School of Chinese Materia Medica, Guangzhou University of Chinese Medicine, Guangzhou, China;; b Dongguan Mathematical En-gineering Academy of Chinese Medicine, Guangzhou University of Chinese Medicine, Guangdong Dongguan, China

**Keywords:** Complete chloroplast genome, *Dendrobium officinale* ‘zhong ke IV hao’, single-molecule real-time (SMRT), PacBio

## Abstract

Here, we reported and characterized the complete chloroplast (cp) genome sequence of *Dendrobium officinale*‘ zhong ke IV hao’, a new variety from self-cross plants of imported Sichuan *D. officinale*, obtained exclusively using Illumina and PacBio sequencing technology. The genome size is 152,185 bp, containing a large single copy (LSC) region (85,094 bp) and a small single copy(SSC) region (14,521 bp) that were separated by two inverted repeat (IRs) regions (26,285 bp).The GC content was 37.46%. In total, the complete cp DNA contains 89 protein-coding genes, 30 tRNA genes, and 8 rRNA genes. Twelve genes contained one or two introns. Phylogenetic analyses showed that the chloroplast genome of *D officinale ‘zhong ke IV hao’* is related to that of the traditional *D.officinale*.

The complete chloroplast (cp) genome is commonly a typical circular double-stranded DNA molecule which has been considered as a super-barcode (Li et al. 2014). Sequencing and analyzing the cp-gnomes of plants can provide evolutionary information for plant species identification, phylogeny, taxonomy, and ecology. Recently, the Pac Bio platform offers unprecedented sufficient throughput and long reads was recognized as the third generation sequencer, which would provide a potential and reliable sequence. Insufficiently, the Pac Bio long reads are error-prone and cannot be analyzed by traditional assembly programs effectively. Thus, we sequenced complete chloroplast genome of *Dendrobium officinale* ‘zhong ke IV hao’ with both Illumina and Pac Bio next-generation technology, in order to contribute to the current understanding of phylogenetic relationships within *Dendrobium*.

*Dendrobium* is one of the largest genera of Orchidaceae, which is the largest family in angiosperms (Xiang et al. 2013). As *D. officinale*, Kimura et Migo were Endangered and often adulterated with other cheaper and more common orchids (Pei Yang et al. 2016) for its economic and medicinal value. In order to solve the problems of wild *D. officinale* in short supply, *D. officinale* ‘zhong ke IV hao,’ which has the characteristics of high production, strong resistance, good quality, and great market potential was cultivated of wild Sichuan *D. officinale*.

Genomic DNA was extracted from fresh leaves of *D.officinale* ‘zhong ke IV hao’ that lives in greenhouse and cultivated by Professor Duan jun from South China Botanical Garden (113.36N, 23.18W), Chinese Academy of Science (Guangzhou, China). We sequenced its genome using Illumina Hiseq4000 (San Diego, CA) and Pac Bio next-generation technology. Genome sequencing was carried out using the Pacific Biosciences RSII (Menlo Park, CA) sequencing platform. Pac Bio long reads (one single-molecule real-time [SMRT] cells, 125 coverage) were assembled using the Hierarchical Genome Assembly Process 2 (HGAP2) protocol from SMRT Analysis version 2.0 package (18), resulting in the complete circular 152,185 bp cp-genome. The cp-genome was assembled using the SOAPdenovo (v2.04) and Celera Assembler (v8.0) and annotated with Dual Organellar GenoMe Annotator (DOGMA) (Wyman et al. 2004). The complete cp-genome sequence was submitted to GenBank under accession number of KX507360.

The chloroplast genome of *D.officinale* ‘zhong ke IV hao’ has a length of 152,185bp and make up of a large single-copy region (LSC, 85,094 bp), a small single-copy region (SSC, 14,521 bp), and two inverted repeat regions (IRs, 26,285 bp). The total GC content is 37.46% while that of the IR regions is 43.0%. This is a little higher than that in the LSC region and the SSC region (35.0% and 30.0%). A total of 127 genes were successfully annotated, including 89 protein-coding genes, 30 tRNA genes, and 8 rRNA genes. The tRNA-coding genes are distributed throughout the genome, 17 in the LSC, one in the SSC, and 12 in the IR regions while rRNAs only situate in IR regions. The portions of protein-coding genes, tRNA genes and rRNA genes are 53.86%, 1.48 %, and 6.92 % in the 152,185bp, respectively. Twelve genes contained one or two introns, containing the protein-coding genes, *atpF*, *clpP*, *ndhB*, *ndhB*, *ndhF*, *rpl2*, *rpl2*, *rpoC1*, *ycf1*, *ycf15*, *ycf15*, and *ycf3*.

The phylogenetic tree of *D.officinale* ‘zhong ke IV hao’ was produced using 17 chloroplast genome sequences of Orchidaceae family which were reported on Genbank (Figure 1). Neighbour-joining analysis was performed with Mega 6. (MEGA Inc., Englewood, NJ) (Tamura et al. 2013), setting model plant *Arabidopsis thaliana* as outgroup and using 500 bootstrap analysis. The bootstrap values were ranged from 59 to 100 in all nodes. As expected *D.officinale* ‘zhong ke IV hao’ is mostly related to traditional *D.officinale* that collected from Zhejiang Province.

**Figure 1.**
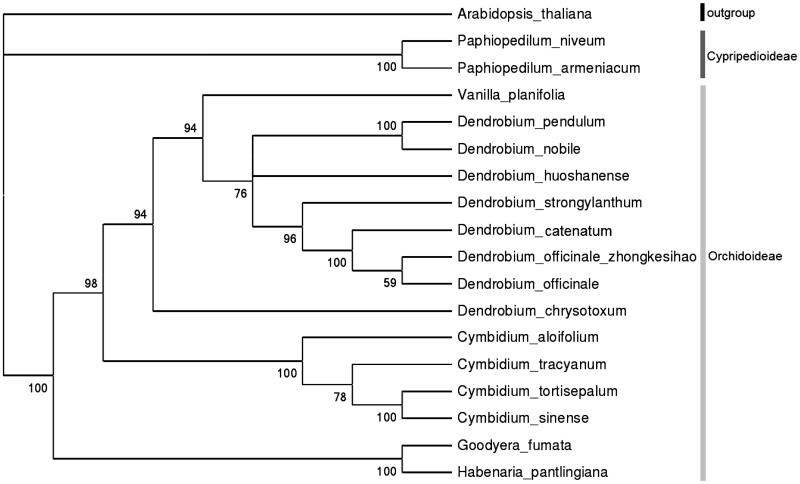
Phylogenetic tree based on neighbour-joining analysis of one outgroup plant and 17 chloroplast genome sequences belonging to Orchidaceae family, which Genbank accession numbers as follows: *A. thaliana* (NC_000932), *Paphiopedilum niveum*(KJ524105), *P. armeniacum* (KJ566307), *Vanilla planifolia* (KJ566306), *D. pendulum* (KT695604), *D. nobile* (KT591465), *D. huoshanense* (KT630834), *D. strongylanthum* (KR673323), *D. catenatum* (KC771275), *D. officinale ‘zhong ke IV hao’*(KX507360), *D. officinale* (KJ862886), *D. chrysotoxum* (KT633996), *Cymbidium aloifolium* (KC876122), *C. tracyanum* (KC876127), *C. tortisepalum* (KC876124), *C. sinense* (KC876123), *Goodyera fumata* (KJ501999), *Habenaria pantlingiana* (KJ524104).
